# Ruptured Sinus of Valsalva Aneurysm and Coarctation of Aorta in a Woman at Early Postpartum Period

**DOI:** 10.1155/2014/731596

**Published:** 2014-03-06

**Authors:** Erol Sener, Aslihan Kucuker, Huseyin Bayram, Kadir Kurt, Emrah Uguz, Muhammed Fethi Saglam

**Affiliations:** ^1^Department of Cardiovascular Surgery, Ataturk Training and Research Hospital, 1426 Cadde, No. 30/17, Cukurambar, 06510 Ankara, Turkey; ^2^Department of Cardiology, Forum Yasam Hospital, 35110 Mersin, Turkey

## Abstract

Coarctation of aorta and sinus of Valsalva aneurysm are frequently missed congenital cardiac defects that their diagnosis might be delayed. To our knowledge, coincidence of these cardiac defects is unusual and has not been reported in the literature before. Here, we present a patient with coarctation of aorta and ruptured noncoronary sinus of Valsalva aneurysm leading to aorto-right atrial fistula in the early postpartum period and our management of this unusual case.

## 1. Introduction

Coarctation of aorta (CoA) is a relatively common abnormality that occurs in approximately 6–8% of patients with congenital heart disease [1]. The diagnosis of coarctation of the aorta may be missed unless an index of suspicion is maintained, and diagnosis is often delayed until the patient develops symptoms. Early diagnosis is possible in the first years of life in symptomatic patients whereas it might be delayed in asymptomatic patients. Untreated CoA might cause severe hypertension, intracranial bleeding, aortic aneurysm formation, and even aortic rupture. Additionally, infective endocarditis, early atherosclerosis due to intimal proliferation and degeneration in coronary arteries may be seen as well.

Sinus of Valsalva aneurysm (SVA) is usually referred to as a rare congenital anomaly. Its origin may be either acquired or congenital. A congenital SVA is usually clinically silent but may vary from a mild, asymptomatic dilatation to symptomatic presentations related to the compression of adjacent structures or intracardiac shunting caused by rupture of the SVA into the right side of the heart. Approximately 65–85% of SVAs originate from the right sinus of Valsalva and the most common complication is rupture into the atrium or ventricle. However, the potential risk of rupture, cardiac failure, stroke, and sudden death has led authors to consider surgical repair of unruptured aneurysms even if they are asymptomatic or incidentally detected, reporting a generally low early surgical and long term mortality [2].

In this case, we report surgical management of a patient in the early postpartum period with a noncoronary SVA complicated with a aorta-right atrial fistula and accompanying asymptomatic and untreated CoA.

## 2. Case History

**Figure 1:**
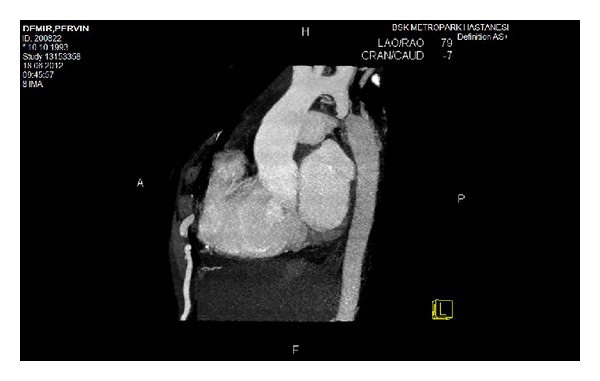
Sinus of Valsalva aneurysm, coarctation of aorta, and dilated internal thoracic artery in CT angiography.

A 19-year-old patient without previous history of cardiovascular disease was admitted to our hospital with chest pain and dyspnea. She had delivered a premature baby one month before her admission. Physical examination revealed 3/6 continuous systolic murmur in aortic valve region. Lower extremity pulses were absent. Blood pressure was 150/60 mm Hg on the right upper extremity and 160/70 mm Hg on the left upper extremity. Laboratory findings were normal except hemoglobin level that was 9.9 g/dL. Transthoracic echocardiography revealed SVA of the noncoronary sinus with a 0.7 cm defect. A flow jet through this defect and a mild right atrial dilatation were observed. Computerized Tomographic Angiography (CTA) confirmed the defect in noncoronary sinus with passage of contrast medium to right atrium and revealed CoA one cm below the orifice of left subclavian artery coincidentally (Figure 1). In order to obtain better images, an aortography was performed showing SVA and aorto-right atrial fistula. Cardiac catheterization revealed 80 mm Hg pressure gradient across the coarctation and Qp/Qs was 1.65.

The patient was scheduled for a staged procedure for these pathologies. In order to reduce the afterload, CoA was repaired with an 18 mm polyester graft interposition through left posterolateral thoracotomy primarily. After one day of intensive care unit (ICU) stay, the patient was discharged in the fifth postoperative day uneventfully.

One month after the first operation, the second stage was performed via median sternotomy for noncoronary SVA and aorto-right atrial fistula repair. The repair was accomplished through standard aortotomy and right atriotomy incisions on cardiopulmonary bypass. The right atrial end of the fistula was closed with interrupted 4-0 polypropylene sutures. Unlike right atrial end, autogenous pericardial patch was used for the closure of the aortic end of the fistula. After one day of ICU stay, the patient was discharged on the sixth postoperative day.

## 3. Discussion

Concomitance of CoA and aorto-right atrial fistula due to SVA rupture is rare. To our knowledge this is the first case of CoA and aorto-right atrial fistula due to rupture of noncoronary SVA following pregnancy.

CoA during pregnancy may cause neonatal or maternal problems due to placental and distal organ hypoperfusion. Poorly controlled hypertension and uterine hypoperfusion may lead to serious adverse neonatal outcomes as growth retardation, premature delivery, and abruptio placenta and/or maternal events as renal failure, hypertensive crisis, or intracranial hemorrhage [3]. In our case, the patient delivered a preterm and low birth weight baby without a predisposing factor other than her yet unknown cardiovascular condition.

SVA is usually referred to as a rare congenital anomaly. Approximately 65–85% of SVAs originate from the right sinus of Valsalva, while SVAs originating from noncoronary (10–30%) and left sinuses (<5%) are exceedingly rare. Congenital SVA is caused by dilation, usually of a single sinus of Valsalva. Other processes that involve the aortic root (e.g., atherosclerotic aneurysms, syphilis, endocarditis, cystic medial necrosis, and chest trauma) may also produce SVA, although this usually involves multiple sinuses. Rupture of the dilated sinus may lead to intracardiac shunting when a communication is established with the right atrium (10%) or directly into the right ventricle (60–90%). It is more prevalent in Asian series [4]. To our knowledge, there are few documented cases of a ruptured SVA during pregnancy or at early postpartum period [5]. Successful vaginal delivery of a full term neonate in a patient with SVA rupture into the right ventricle causing left to right shunting had been reported in the literature [6]. In our case, the diagnosis was made in the early postpartum period and the time of rupture is unclear. We assumed that cardiovascular condition in pregnancy and/or exertion during delivery had triggered SVA rupture.

In an uncomplicated pregnancy, plasma volume increases up to 45% and total blood volume increases almost 40%. Parameters such as heart rate, stroke volume, and cardiac output also increase. Therefore, pregnancy is a state that imposes hemodynamic strain on the cardiovascular system and might be a risky period in patients with underlying cardiac disease. Hypertensive complications such as aortic rupture and dissection in pregnancy are more common in women with CoA [7]. In previous studies, aortic rupture and dissection have been reported in women with repaired and unrepaired CoA [8]. In our case, uncontrolled hypertension might have evoked rupture.

Acquired aorto-atrial fistulas, which seem to be more common than the congenital form, have been described in association with prosthetic valve endocarditis, penetrating or blunt chest trauma, aortic dissection, and ventricular septal defect repair [4, 9]. Recent reports have examined transcatheter closure of ruptured SVA and complications such as residual shunts, device migration, and haemolysis were published [9].

CoA stenty might also result in serious complications as fatal vessel rupture, pseudoaneurysm formation, stent migration, and sustained hypertension [10]. Therefore, in this case, we performed staged surgical repair of CoA and aorta-right artial fistula. The advantages of this staged approach are decreasing the left to right shunt exacerbated by CoA, reducing the risk of one stage complex approach, and closure of the fistula under direct control. The optimal surgical or interventional management of this patient is a matter of debate but we preferred a multistaged surgical strategy via two different thoracic incisions at different times.
